# Pembrolizumab vs cemiplimab for the treatment of advanced non-small cell lung cancer with PD-L1 expression levels of at least 50%: A network meta-analysis and cost-effectiveness analysis

**DOI:** 10.3389/fonc.2022.878054

**Published:** 2022-09-26

**Authors:** Yan Li, Xueyan Liang, Tong Yang, Sitong Guo, Xiaoyu Chen

**Affiliations:** ^1^ Department of Pharmacy, The People’s Hospital of Guangxi Zhuang Autonomous Region, Nanning, China; ^2^ School of Pharmaceutical Sciences, Guangxi Medical University, Nanning, China

**Keywords:** cost-effectiveness, non-small lung cancer, pembrolizumab, cemiplimab, network meta-analysis

## Abstract

**Background:**

Pembrolizumab and cemiplimab have been approved as treatment for advanced non-small-cell lung cancer (NSCLC) with high programmed death ligand-1 (PD-L1) expression. This study aimed to evaluate the cost-effectiveness of pembrolizumab compared with that of cemiplimab in the treatment of advanced NSCLC with high PD-L1 expression from a societal perspective in the United States.

**Materials and methods:**

Cost-effectiveness analysis integration of the network meta-analysis framework was performed using data from the EMPOWER-Lung 1, KEYNOTE 024, and KEYNOTE 042 phase 3 randomized clinical trials. A network meta-analysis including 2289 patients was constructed, and the Markov and partitioned survival (PS) models were used to assess the cost-effectiveness of pembrolizumab compared with that of cemiplimab for the treatment of high PD-L1 expression (≥50% of tumor cells). The time horizon was 10 years. The main outcomes were overall costs, incremental cost-effectiveness ratios (ICERs), quality-adjusted life-years (QALYs), life-years, incremental net health benefits (INHB), and incremental net monetary benefits (INMB). The robustness of the model was verified using one-way and probabilistic sensitivity analyses, and subgroup analyses were conducted.

**Results:**

Treatment of advanced NSCLC with high PD-L1 expression with pembrolizumab achieved 0.093 QALYs and was associated with an incremental cost of $10,657 compared with cemiplimab, yielding an ICER of $114,246/QALY. The ICER in the PS model was similar to that in the Markov model, with a difference of $3,093/QALY. At a willingness-to-pay (WTP) threshold of $100,000/QALY, INHB, and INMB of pembrolizumab were -0.013 QALYs and -$1,329, respectively, and the probability of cemiplimab was 51% when compared with pembrolizumab. When the WTP threshold increased to $150,000/QALY, the INHB and INMB of pembrolizumab were 0.022 QALYs and $3,335, respectively, and the probability of pembrolizumab was 51.85%. One-way sensitivity analysis indicated that the models were sensitive to pembrolizumab and cemiplimab costs. Subgroup analysis revealed that treatment with pembrolizumab was related to a higher INHB in several subgroups, including patients with brain metastases at baseline.

**Conclusion:**

Our findings suggest that the WTP threshold should be considered when choosing between cemiplimab and pembrolizumab to treat advanced NSCLC with high PD-L1 expression. Reducing the cost of pembrolizumab may lead to valuable outcomes.

## Introduction

Lung cancer is one of the most common types of carcinomas and the leading cause of cancer death (1), causing nearly 1.8 million deaths (18%) worldwide (2). Because lung cancer is often diagnosed at an advanced stage, its prognosis is poor. Lung cancer is generally divided into four histological categories, with non-small cell lung cancer (NSCLC) accounting for 85-90% of all lung cancers (3, 4). Lung squamous and non-squamous cell carcinomas constitute 25-30% and 70-75% of NSCLC cases, respectively (1, 5). Progression to advanced or metastatic cancer occurs in approximately 50% of NSCLC cases (1, 6, 7). Similarly, in patients with local or locoregional NSCLC, a high proportion deteriorate into recurrent or metastatic NSCLC (8–10), and the prognosis in patients with distant metastatic NSCLC remains poor, with the 5-year survival data reported to be approximately 5% (1). Based on the current situation, new effective treatments for NSCLC are urgently required.

In recent years, newer and more effective therapies, such as immune checkpoint inhibitors (ICIs), have been used for the treatment of patients with NSCLC, which has gradually improved treatment regimens for patients with advanced NSCLC (11). With elevated neo-antigen expression levels and high immune evasion of tumor cells, lung cancer presents an ideal setting for the expression of programmed cell death-1 (PD-1), programmed death-ligand 1 (PD-L1), and Cytotoxic-T-lymphocyte-antigen-4 (CTLA-4). It is estimated that 25% of patients with advanced NSCLC exhibit high PD-L1 expression (12, 13). In recent years, ICIs have shown superiority over chemotherapy; however, they do not rely on PD-L1 expression levels (14); therefore, evidence of high PD-L1 expression to promote the utility of immunotherapy of PD-L1/PD-1 is limited.

Cemiplimab (15, 16) is a humanized recombinant monoclonal antibody that blocks high affinities (15). In September 2018, the US Food and Drug Administration approved cemiplimab for the treatment of metastatic or locally advanced cutaneous squamous cell carcinoma (CSCC), because of its strong antitumor activity and high safety (17, 18). Pembrolizumab, which binds to PD-1 to inhibit tumor growth, is the current preferred treatment for metastatic NSCLC and was approved for marketing by the European Commission in January 2017. It is a human immunoglobulin (Ig) G4 monoclonal antibody with affinity and high selectivity. This treatment is targeted to populations with high PD-L1 expression, and no epidermal growth factor receptor (EGFR) mutations or anaplastic lymphoma kinase (ALK) translocations (19). Overall, pembrolizumab and cemiplimab are attractive first-line immunotherapy options for patients with advanced NSCLC.

Cemiplimab and pembrolizumab have both been approved for the treatment of advanced NSCLC, and have similar efficacy. However, their high costs make them unaffordable for a considerable proportion of patients with advanced NSCLC, with many having to choose to give up or postpone treatment, reduce their quality of life, or even face bankruptcy (20–23). For clinicians, patients, and decision makers, it is extremely important to evaluate the cost-effectiveness of therapeutic strategies to make health decisions and to allow the optimal allocation of limited health resources. This makes cost-effectiveness evaluations highly important. As such, this study was designed to evaluate the cost-effectiveness of pembrolizumab compared with that of cemiplimab as the preferred first-line treatment for advanced NSCLC with high PD-L1 expression.

## Methods

### Network meta-analysis

#### Search strategy, selection of studies and quality assessment

We systematically searched the PubMed, Medline (*via* OVID SP), Embase (*via* OVID SP), and Cochrane CENTRAL databases from the time of their inception to November 28, 2021. The search was performed without the limitation of restrictions on publication year or language. Details of the study selection process are shown in **Supplementary Figure 1**. Quality assessment of the included studies was performed by Li and Liang, according to the Cochrane risk-of-bias tool (24).

### Statistical analysis

Network meta-analysis was performed using the netmeta package in R, version 4.0.2 (The R Foundation for Statistical Computing) to obtain the hazard ratios (HRs) with 95% confidence interval (95% CI) for overall survival (OS) and progression-free survival (PFS) between pembrolizumab and cemiplimab. A fixed-effects model was used.

### Cost-effectiveness analysis

#### Analytical overview

We performed a cost-effectiveness analysis comparing pembrolizumab with cemiplimab. The patients cohort was obtained from the randomized clinical trials (RCTs) EMPOWER-Lung 1 (25), KEYNOTE 024 (26), and KEYNOTE 042 (27).

This cost-effectiveness analysis was performed according to the Consolidated Health Economic Evaluation Reporting Standards (CHEERS) guidelines (28). This study did not use individual patient data and did not include human or animal research; hence, an institutional review board or ethics committee approval was not required for this study, according to the guidelines of the US Department of Health and Human Services (45 CFR §46) (29).

#### Model structure

**Figure 1:**
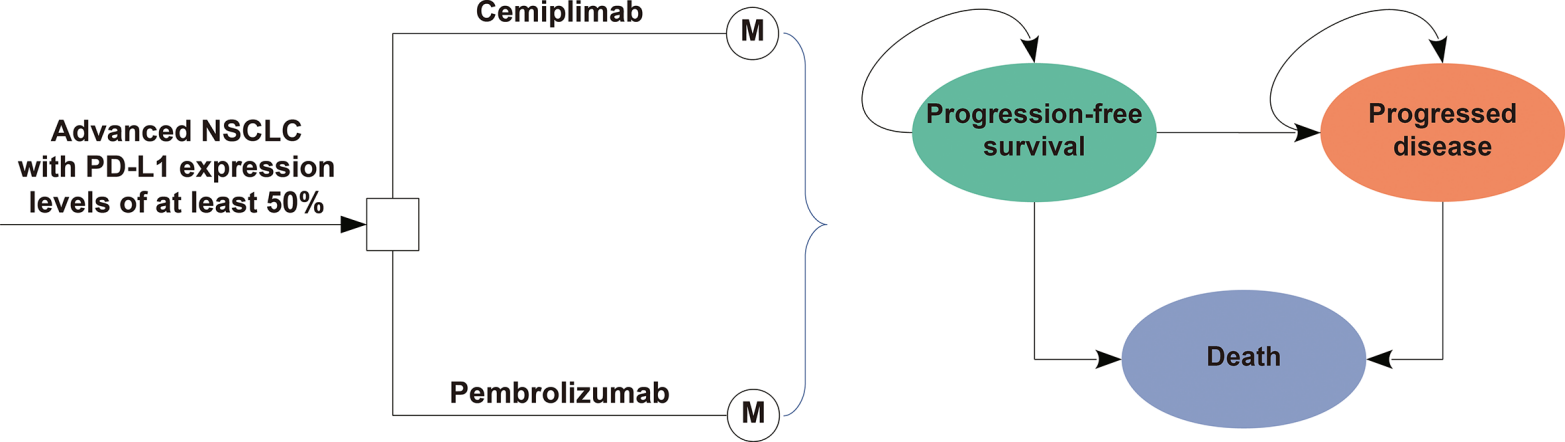
Model Structure of a Decision Tree Combining the Markov Model with the three Health States.

Markov model and partitioned survival (PS) models were constructed to assess the outcomes of pembrolizumab versus cemiplimab in the therapy of advanced NSCLC with high PD-L1 expression from the societal perspective in the United States (US). The model included three health states: PFS, progressed disease (PD), and death (**Figure 1**). The cycle length for the Markov and PS models was one week, and the time horizon was 10 years. During each cycle, the patients either remained in their existing health state or progressed to the next health state.

The main outcomes of our study were overall costs, incremental cost-effectiveness ratios (ICERs), quality-adjusted life-years (QALYs), life-years, incremental net health benefits (INHB), and incremental net monetary benefits (INMB).

#### Clinical data inputs

The OS and PFS survival curves of patients in the pembrolizumab and cemiplimab groups were obtained from the EMPOWER-Lung 1 (25), KEYNOTE 024 (26), and KEYNOTE 042 (27) trials, and data beyond the trial follow-up time horizon were generated using an algorithm created by Guyot et al. (30). The EMPOWER-Lung 1 trial is an open-label, phase 3 RCT, performed between June 27, 2017, and February 27, 2020, which evaluated the survival efficacy and safety of cemiplimab versus platinum-based chemotherapy in the treatment of patients with NSCLC with PD-L1 expression levels of at least 50%. The other two open-label, phase 3 RCTs, KEYNOTE-024 and KEYNOTE-042, evaluated the survival efficacy and safety of pembrolizumab compared to platinum-based chemotherapy in patients with NSCLC (26, 27). KEYNOTE-024 was conducted between September 19, 2014, and July 10, 2017, and KEYNOTE-042 was conducted between June Dec 19, 2014, and March 6, 2017.

The Kaplan-Meier survival curves of PFS and OS data were obtained from the three trials using GetData Graph Digitizer version 2.26 to extract the individual patient data points, and subsequently, six parametric survival models, Weibull, log-normal, log-logistic, exponential, generalized gamma, and Gompertz were used to fit these data. The appropriate survival model was selected according to the lowest Akaike and Bayesian information criteria. The survival model results of the cemiplimab and pembrolizumab groups are shown in **Table 1**, and the results of a good visual fit to the data are shown in **Supplementary Table 1**. The survival events and survival times of virtual individual patients were similar to the practical number at risk, which closely reappeared in the survival curves. Additional results of model fitting are shown in detail in **Supplementary Figure 2**. After disease progression, second-line treatment schemes were recorded from the EMPOWER-Lung 1, KEYNOTE 024 and KEYNOTE 042 trials. Primary clinical inputs are listed in **Table 1**.

**Table 1:** Key model inputs.

Parameter	Value (95% CI)	Distribution	Source
Lognormal OS survival model of cemiplimab^a^	μ = 4.97513 σ = 1.78373	Lognormal	Model fitting
Lognormal PFS survival model of cemiplimab^a^	μ = 3.45945 σ = 1.29574	Lognormal	Model fitting
Log-logistic OS survival model of pembrolizumab^a^	μ = 0.98918 σ = 83.81104	Lognormal	Model fitting
Lognormal PFS survival model of pembrolizumab^a^	μ = 3.42683 σ = 1.42989	Lognormal	Model fitting
HR for OS (cemiplimab vs pembrolizumab)	0.85 (0.60 to 1.20)	Lognormal	Network meta-analysis
HR for PFS (cemiplimab vs pembrolizumab)	0.67 (0.49 to 0.90)	Lognormal	Network meta-analysis
Body surface area, m^2^	1.86 (1.40 to 2.23)	Gamma	Pei 2021 (29)
Body weight, kg	70 (50 to 91)	Gamma	Pei 2021 (29)
**Drug costs per 1 mg** ^b^			
Price of cemiplimab	27.54 (20.66 to 34.43)	Gamma	CMS (31)
Price of pembrolizumab	52.75 (39.57 to 65.94)	Gamma	CMS (31)
Price of gemcitabine	0.02 (0.01 to 0.02)	Gamma	CMS (31)
Price of paclitaxel	0.13 (0.1 to 0.16)	Gamma	CMS (31)
Price of cisplatin	0.18 (0.13 to 0.22)	Gamma	CMS (31)
Price of pemetrexed	7.49 (5.62 to 9.36)	Gamma	CMS (31)
Price of carboplatin	0.05 (0.04 to 0.07)	Gamma	CMS (31)
Second-line treatment in cemiplimab arm per cycle (total 18 cycles)^c^	1332.92 (999.69 to 1666.15)	Gamma	CMS (31)
Second-line treatment in pembrolizumab arm per cycle (total 18 cycles)^d^	30.09279(22.57 to 37.62)	Gamma	CMS (31)
Cost of terminal care per patient^*^	16441.83 (12331.37 to 20552.29)	Gamma	Insinga et al, 2019 (32)
**Drug administration cost**			
First hour	148.3 (111.23 to 185.38)	Gamma	CPT code 96413 (31)
Additional hour	31.4 (23.55 to 39.25)	Gamma	CPT code 96415 (31)
**Cost of managing AEs (grade ≥ 3)^e^**			
Pembrolizumab	1051.76 (788.82 to 1314.7)	Gamma	Konidaris et al, 2020 (33); Wong et al, 2018 (34)
Cemiplimab	440.22 (330.17 to 550.275)	Gamma	Konidaris et al, 2020 (33); Wong et al, 2018 (34)
**Disease costs per cycle**			
Stable disease	464.85 (348.64 to 581.06)	Gamma	Insinga et al, 2019 (32)
Progressed disease	1075.49 (806.62 to 1344.36)	Gamma	Insinga et al, 2019 (32)
**Societal costs per cycle**			
Patient time and salary loss	134.22 (100.66 to 167.77)	Gamma	Guérin et al, 2016 (35)
Parking, meals, and travel	11.33 (0.97 to 22.71)	Gamma	Lauzier et al, 2011 (36)
Caregiver	160.95 (119.01 to 226.68)	Gamma	Li et al, 2013 (37)
**Health utilities**			
**Disease status utility per year**			
Utility of PFS	0.754 (0.407 to 0.970)	Beta	Nafees et al, 2017 (38)
Utility of PD	0.180 (0.115 to 0.367)	Beta	Nafees et al, 2017 (38)
Death	0	NA	
**Drug toxic effects disutility^f^**			
Pembrolizumab	0.0192 (0.0144 to 0.024)	Beta	Nafees et al, 2017 (38) Freeman et al, 2015 (39)
Cemiplimab	0.0083 (0.0062 to 0.0104)	Beta	Nafees et al, 2017 (38); Freeman et al, 2015 (39)

AE, adverse event; HR, hazard ratio; OS, overall survival; PD, progressed disease; PFS, progression-free survival.

^a^Only expected values are presented for these survival model parameters.

^b^Costs are in 2021 US dollars and adjusted for inflation as appropriate, and average sale price plus 4.2% to calculate drug costs.

^c^Calculated as the average cost of treatment using weighted frequencies of individual second-line therapeutic agents received by each treatment arm in the EMPOWER-Lung 1 trial.

^d^Calculated as the average cost of treatment using weighted frequencies of individual second-line therapeutic agents received by each treatment arm in the KEYNOTE 024 and KEYNOTE 042 trials. ^e^Calculated as the average cost of toxic effects using weighted frequencies of grade ≥ 3 treatment related adverse events for each treatment arm in the EMPOWER-Lung 1, KEYNOTE 024 and KEYNOTE 042 trials. Costs of individual toxic effects were derived from the literature and include all care required to manage each toxic effect. References for individual toxic effect costs are summarized in **Supplementary Table 2**. ^f^Calculated as the average disutility of toxic effects using weighted frequencies of grade ≥ 3 treatment-related adverse events for each treatment arm in the EMPOWER-Lung 1, KEYNOTE 024 and KEYNOTE 042 trials. Disutility from experiencing toxic effects occurred over a 1-month period. Disutilities of individual toxic effects were derived from the literature. References for individual toxic effect disutilities are summarized in **Supplementary Table 2**.

#### Cost and utility inputs

In performing this analysis, we considered the direct medical and societal perspective costs. Direct medical costs included the costs of drugs, those due to the health state of the patient, those for the management of adverse events (AEs) related to toxic effects, and those for terminal care (**Table 1**). In addition to direct medical costs, our societal perspective model incorporated informal health care costs, such as patient time and/or salary (35), transportation (36), and caregiver (37) costs. All costs were adjusted to 2021 US dollars, and inflation was calculated using Medical-Care Inflation data obtained from Tom’s Inflation Calculator (40), the values of which are shown in **Table 1** (31–37, 39). Based on the EMPOWER-Lung 1 trial report, patients treated with cemiplimab received cemiplimab 350 mg every 3 weeks, and according to the KEYNOTE 024 and KEYNOTE 042 trials reports, patients in the pembrolizumab group received pembrolizumab 200 mg intravenously every 3 weeks. Both arms received pembrolizumab or cemiplimab for up to 108 weeks until disease progression, unacceptable toxicity, or death. To calculate direct drug costs, costs of drugs were acquired from the average sale price of 2021 from the Centers for Medicare and Medicaid Services (CMS), adding 4.2% to estimate the current drug price (31). To estimate the dosage of second-line chemotherapy, we assumed that the body surface area of a typical patient is 1.86 m^2^, and the body weight is 70 kg (29). The costs associated with monitoring PFS and PD stage patients were $465 per cycle and $1,075 per cycle, respectively (32). The cost of terminal care was $16,441.83 per patient with advanced NSCLC (32). This analysis estimated the costs of managing grade ≥ 3 treatment-emergent AEs obtained from literature (**Supplementary Table 2**).

Each NSCLC health state was related to a preference-based health utility on a scale of 0 (death) to 1 (perfect health). The PFS and PD states related to advanced NSCLC were 0.754 and 0.18 (38), respectively. In this analysis, disutility was considered as AEs of grade 3 or higher.

### Base-case analysis

The ICER was expressed as the cost per additional QALY gained between pembrolizumab and cemiplimab. When the ICER is smaller than a predefined WTP threshold, cost-effectiveness is indicated (41). Given the evidence suggesting that $50,000 per QALY is too low in the US, this might best be thought of as an implied lower boundary, and a willingness-to-pay (WTP) threshold of $100,000 to $150,000 per QALY was therefore set (41). Costs and utility outcomes were discounted at 3% per year (42). The INHB and INMB were calculated using the following formulas:


$$\text{INHB}\left(\lambda \right)=\left(\mu_{Ep}-\mu_{Ec}\right)-\frac{\mu_{Cp}-\mu_{Cc}}{\lambda }=\;\Delta E-\Delta C/\lambda$$

and


$$\text{INMB}\left(\lambda \right)=\left(\mu_{Ep}-\mu_{Ec}\right)\times \lambda -(\mu_{Cp}-\mu_{Cc})=\;\Delta E\times \lambda -\Delta C,$$

where μ*_E_*_p_ and μ*_E_*_c_ are the effectiveness of pembrolizumab and cemiplimab, respectively; μ*_C_*_p_, μ*_C_*_c_ and are the costs of pembrolizumab and cemiplimab, respectively; and λ is the WTP threshold (43, 44).

### Sensitivity and subgroup analyses

In this study, we performed one-way and probabilistic sensitivity analyses to estimate the robustness of the model outcomes. One-way sensitivity analyses were performed for different variables, including costs and utilities, and the uncertainty of each variable was either calculated according to the 95% CIs reported in the literature, or estimated by assuming a 25% variation from the baseline values (**Table 1**). A probabilistic sensitivity analysis with 10,000 iterations was performed using Monte Carlo simulation to test the uncertainty of the model with pre-specified probability distributions. Gamma distribution was used for the cost parameters, log-normal distribution was used for the HRs, and beta distribution was selected for the probability, proportion, and preference value parameters. A cost-effectiveness acceptability curve was drawn to evaluate the possibility that pembrolizumab or cemiplimab would be valuable at different WTP values for QALY gains according to the results obtained from 10,000 iterations. Subgroup analyses were constructed for the subgroups reported in the trials of EMPOWER-Lung 1, KEYNOTE 024, and KEYNOTE 042 using different HRs for PFS and OS. All statistical analyses in this study were performed using the hesim and heemod packages in R, version 4.0.5, 2021 (R Foundation for Statistical Computing).

## Results

### Network meta-analysis

The database search identified 1,215 records, and three phase 3 RCTs (EMPOWER-Lung 1, KEYNOTE 024, and KEYNOTE 042) involving 2289 patients were evaluated in the network meta-analysis (**Supplementary Figure 3**). In the EMPOWER-Lung 1 trial, 710 patients were allocated to the cemiplimab or platinum-based chemotherapy group; in KEYNOTE 024, 305 patients were assigned to receive pembrolizumab or platinum-based chemotherapy; while in KEYNOTE 042, 1,274 patients received pembrolizumab or platinum-based chemotherapy treatment. The risk of bias and methodological quality of the studies were evaluated, and are shown in **Supplementary Figure 4**. The network meta-analysis revealed that compared with cemiplimab, the HRs for OS and PFS of pembrolizumab was 1.18 (95% CI, 0.83-1.67) and 1.49 (95% CI, 1.11-2.04), respectively.

### Cost-effectiveness analysis

#### Base-case analyses

Compared with cemiplimab, pembrolizumab provided an additional 0.093 QALYs with an additional cost of $10,657, which was related to an ICER of $114,246/QALY in the Markov model. The INHB was -0.013 and 0.002 QALYs, and the INMB was -$1,329 and $3,335 at WTP thresholds of $100,000/QALY and $150,000/QALY, respectively (**Table 2**). We found that the ICER in the PS model was similar to that in the Markov model, with a difference of $3,093/QALY (**Table 2**). On the other hand, compared with platinum-based chemotherapy, the corresponding ICERs of pembrolizumab and cemiplimab were $175,442/QALY and $211,130/QALY, respectively (**Supplementary Table 3**).

**Table 2:** Summary of cost and outcome results in the base-case analysis in the markov model and partitioned survival model.

Factor	Cemiplimab	Pembrolizumab	Incremental pembrolizumab vs cemiplimab
**Markov model**			
Cost, $			
First-line drug	104,883	143,114	38,232
Disease costs	112,046	93,088	-18,958
Drug administration cost	3,478	3,300	-178
Overall	271,957	282,613	10,657
Life-years			
Progression-free	0.715	0.966	0.251
Overall	2.637	2.394	-0.243
QALYs	0.826	0.920	0.093
Incremental cost per QALY^a^	114,246		
INHB, QALY, at WTP threshold 100000^a^	-0.013		
INMB, $, at WTP threshold 100000^a^	-1,329		
INHB, QALY, at threshold 150000^a^	0.022		
INMB, $, at threshold 150000^a^	3,335		
**Partitioned Survival Model**			
Cost, $			
First-line drug	106,958	144,990	38,032
Disease costs	111,522	92,683	-18,839
Drug administration cost	146,431	199,898	53,468
Overall	272,656	284,071	11,414
Life-years			
Progression-free	0.891	1.567	0.676
Overall	2.542	2.389	-0.153
QALYs	0.833	0.931	0.097
Incremental cost per QALY^a^	117,339		
INHB, QALY, at WTP threshold 100000^a^	-0.017		
INMB, $, at WTP threshold 100000^a^	-1,687		
INHB, QALY, at threshold 150000^a^	0.021		
INMB, $, at threshold 150000^a^	3,177		

INHB, incremental net health benefit; INMB, incremental net monetary benefit; QALY, quality-adjusted life-years.

^a^Compared with cemiplimab.

### Sensitivity analysis

**Figure 2:**
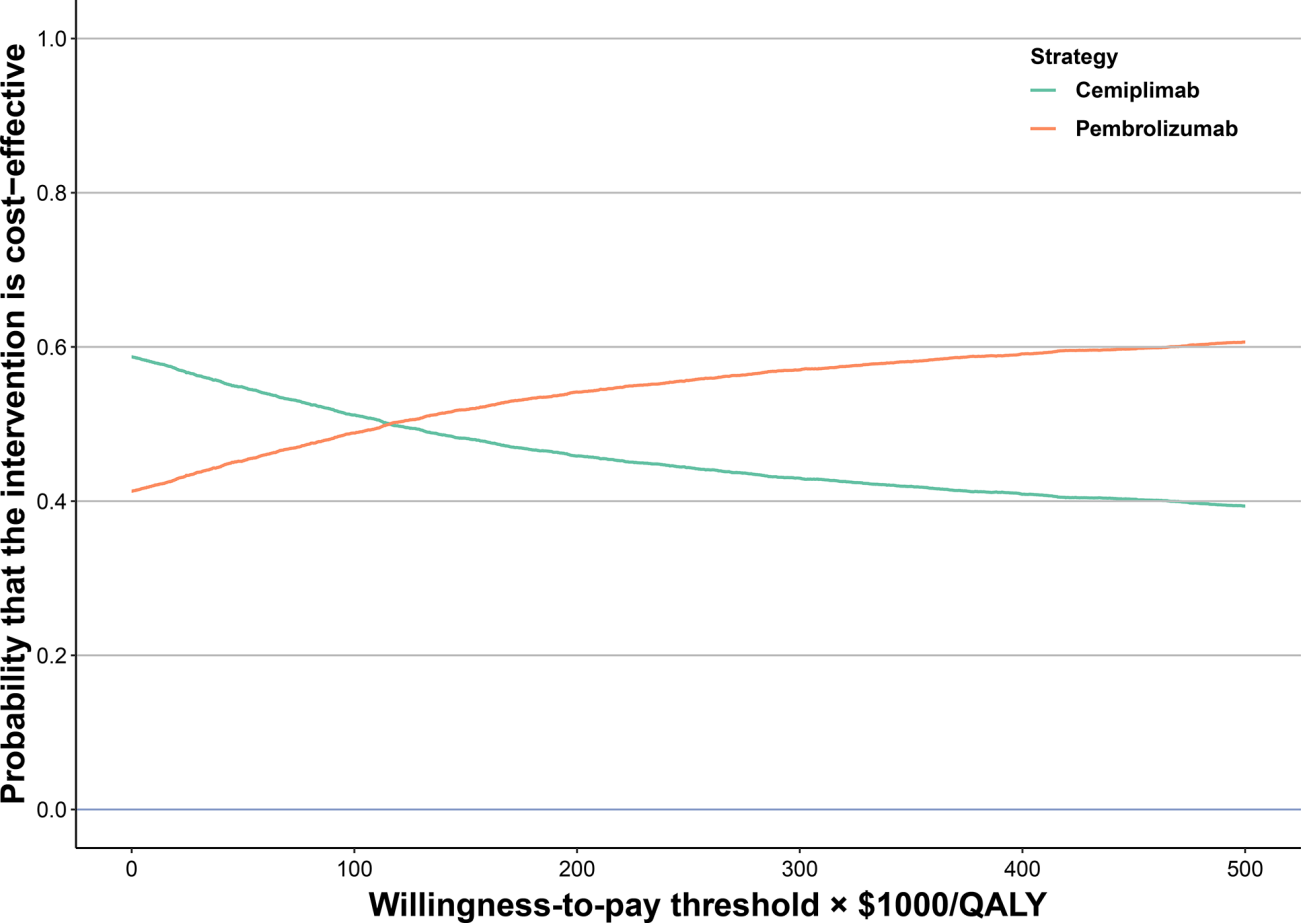
Cost-effectiveness Acceptability Curves for Pembrolizumab vs Cemiplimab.

One-way sensitivity analyses illustrated that the primary drivers of the model outcome were the cost of pembrolizumab and cemiplimab (**Supplementary Figure 5**) because this factor had the greatest impact on ICER. The model results were robust to the uncertainty of other model inputs, such as the cost of second-line chemotherapy therapy, AE related costs, and disutilities. Compared with cemiplimab, the cost-effectiveness acceptability curves revealed that pembrolizumab was associated with a cost-effectiveness probability of 48.82% and 51.85% when the WTP thresholds were $100,000, and $150,000, respectively (**Figure 2**). We subsequently estimated the relevance of these key variables to the ICER between cemiplimab and pembrolizumab. When the WTP threshold was set as $100,000/QALY, pembrolizumab was cost-effective when the cost of pembrolizumab was less than $56.26 per mg or the cost of cemiplimab exceeded $26.88 per mg. When the WTP threshold was increased to $150,000/QALY, and the cost of pembrolizumab less than $53.98 per mg or the cost of cemiplimab was exceeded $26.69 per mg, pembrolizumab was cost-effective; otherwise, cemiplimab was preferable (**Supplementary Figure 6**).

### Subgroup analysis

**Figure 3:**
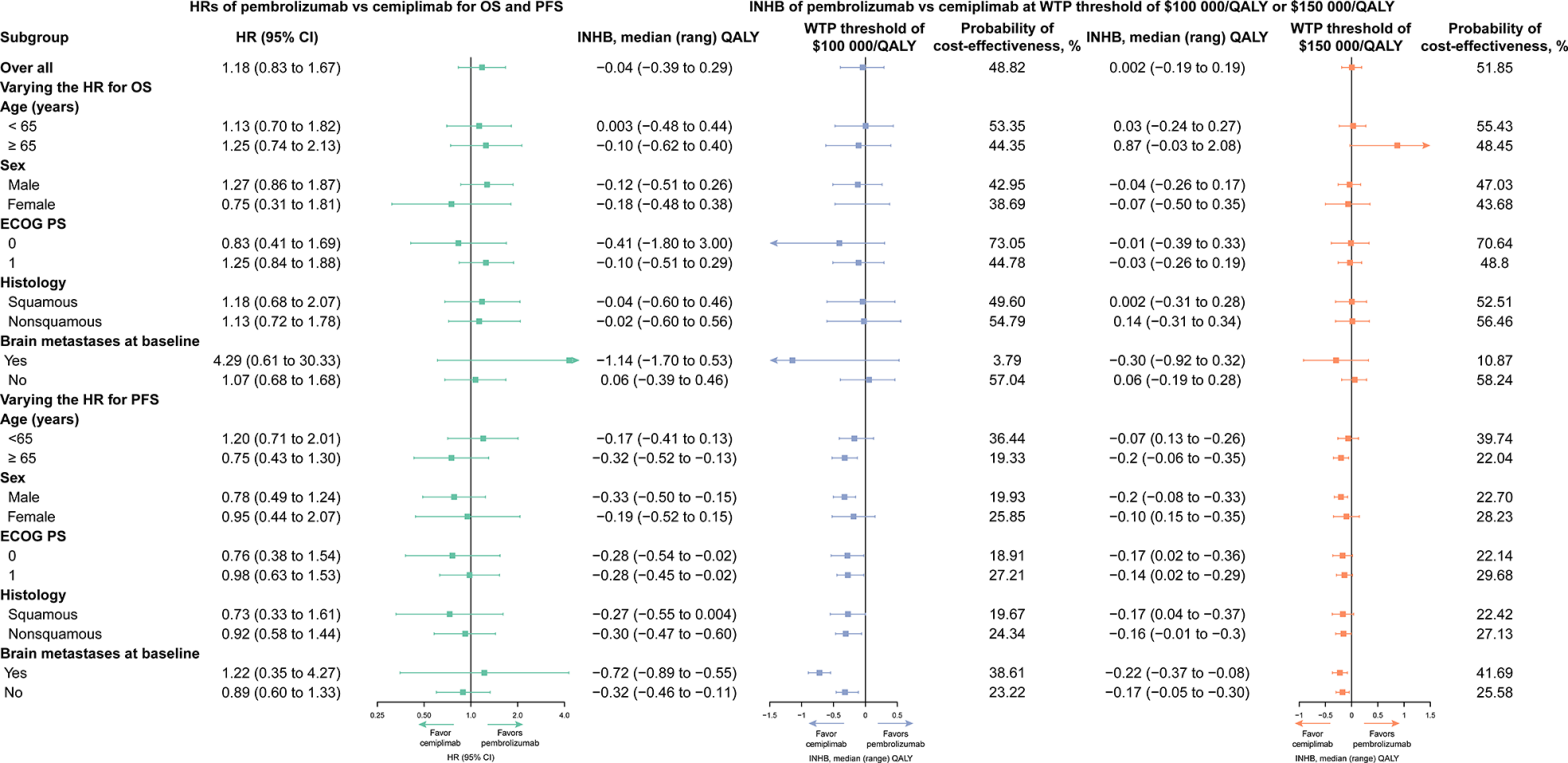
Subgroup Analysis Results of Incremental Net Health Benefits (INHBs) and Probabilities of Cost-effectiveness Obtained by Varying the Hazard Ratios (HRs) for Overall Survival and Progression-free Survival.

In this study, we performed subgroup analyses to evaluate various HRs for OS, and the results showed that pembrolizumab was related to positive INHB values with ≥ 50% probability, and should be considered cost-effective in the following subgroups: patients aged less than 65 years, those with an Eastern Cooperative Oncology Group score of 0, those with non-squamous cell carcinoma, and those without brain metastases at baseline, at either of the $100,000/QALY or $150,000/QALY thresholds (**Figure 3**).

## Discussion

In this study, we performed a network meta-analysis and cost-effectiveness analysis based on the findings of the KEYNOTE 024, EMPOWER-Lung 1, and KEYNOTE 042 trials. Our results revealed that, compared with cemiplimab, pembrolizumab was associated with an incremental survival of 0.093 QALYs and an additional cost of $10,657, which was related to an ICER of $114,246/QALY for the treatment of advanced NSCLC. The cost-effectiveness acceptability curves revealed that the probability of pembrolizumab being more cost-effective was 48.82% and 51.85% at WTP thresholds of $100,000 and $150,000/QALY, respectively. Subgroup analysis results revealed that pembrolizumab was the preferable treatment drug in the majority of the subgroups because of its relation to positive INHBs and higher than 50% possibility of cost-effectiveness compared with cemiplimab at a threshold of $150,000/QALY. The results of this study are robust, as demonstrated by further one-way and probabilistic sensitivity analyses. The one-way sensitivity analysis results suggested that the cost of pembrolizumab and cemiplimab was the most influential factor in patients with advanced NSCLC with high PD-L1 expression; however, the WTP findings were stable when the cost of pembrolizumab and cemiplimab fluctuated within a reasonable range.

Prior studies also evaluated the cost-effectiveness of pembrolizumab (5, 45) or cemiplimab (46) monotherapy for patients with advanced NSCLC with high PD-L1 expression compared to platinum-based chemotherapy. In general, pembrolizumab is the preferred treatment for patients with NSCLC with high PD-L1 expression (26, 27). Georgieva et al. suggested that first-line pembrolizumab for NSCLC may be cost-effective in the US compared to platinum-based chemotherapy, but not in the United Kingdom (UK) (45). More specifically, the ICERs/QALY for the UK and US were $52,000 and $49,000, respectively (45). Christos Chouaid et al. considered that pembrolizumab may be cost-effective for patients treated in France, and the ICER/QALY of pembrolizumab versus platinum-based doublets was $95,870 (5). A previously published paper also compared the cost-effectiveness of cemiplimab versus platinum-based chemotherapy, yielding an ICERs of $40,390/QALY in patients with advanced NSCLC with high PD-L1 expression (46). In this study, we investigated cost-effectiveness from the societal perspective in the US, which could explain why the ICERs of pembrolizumab and cemiplimab were higher than that of in previously published studies compared with platinum-based chemotherapy.

It is worth emphasizing the advantages of this study. First, to our knowledge, this is the first study to assess the cost-effectiveness of pembrolizumab versus cemiplimab for the treatment of advanced NSCLC with high PD-L1 expression from a societal perspective using Markov and PS models. Second, we used a network meta-analysis approach to perform an indirect comparison of the two ICIs. Moreover, we established a 10-year Markov model and PS model, which included sufficient variables to explore the cost-effectiveness of immunotherapies. We also considered the costs from direct medical and societal perspectives. Third, our study analyzed the cost-effectiveness of the 10 subgroups prespecified by the EMPOWER-Lung 1, KEYNOTE 024, and KEYNOTE 042 trials. Cost-effective outcomes for the subgroups may aid psychiatrists, clinicians, and decision-makers to develop appropriate therapeutic strategies for patients with special characteristics. Fourth, we adopted Markov and PS models to perform the cost-effectiveness analysis, and the ICER in the PS model was similar to that in the Markov model.

This study has some limitations which should be mentioned. First, although the EMPOWER-Lung 1, KEYNOTE 024, and KEYNOTE 042 trials similarly focused on advanced NSCLC with high PD-L1 expression, there was heterogeneity between them. Interpretations of these results should be performed cautiously because of the potential bias in these three trials that did not provide the original individual patient data. Second, we assumed that the risk of AEs and the percentages of management of AEs in the subgroup of patients were equal to those in the treatment groups. Furthermore, subgroup analysis with a limited sample size decreased the robustness of the model outcomes. Third, the robustness of the model was evaluated by its structure, assumptions, data sources, analyses, and results. We evaluated all uncertainties in the sensitivity analyses. Considering that pembrolizumab and cemiplimab are comparatively new treatment for patients with advanced NSCLC with high PD-L1 expression, long-term follow-up survival data were not reported, and the accuracy of the results of our study could not be further validated and explored. Fourth, in the sensitivity analyses, we assumed a 25% variation in the baseline values of variables, without providing the range of confidence intervals. This assumption method is frequently used in economic assessments; however, this interval may be inaccurate for some variables. Fifth, considering the differences in cost inputs and payment capacity in different regions, the results of this study may not be applicable to other countries (29). Finally, our societal perspective model incorporated informal healthcare costs, such as patient time and/or salary, transportation, and caregiver costs. However, these costs were not evaluated in patients with NSCLC, and may be inaccurate for some variables.

## Conclusions

We performed a network meta-analysis and cost-effectiveness analysis to evaluate the cost-effectiveness of pembrolizumab and cemiplimab for the treatment of patients with advanced NSCLC with high PD-L1 expression from a societal perspective in the US. This economic evaluation found that the optimal therapy choice between pembrolizumab and cemiplimab could be cost-effective for patients with advanced NSCLC with high PD-L1 expression, in consideration of the WTP threshold. Given a WTP of $100,000/QALY, cemiplimab was cost-effective; however, at a WTP threshold of $150,000/QALY, pembrolizumab was cost-effective. Economic evaluation may be improved by adjusting the price of pembrolizumab or cemiplimab. Considering the limitations of our study, additional well-designed, high-quality RCTs and real-world studies are urgently required. We believe that the results of our study will provide clinical evidence and play an important role in the evaluation of the value of pembrolizumab and cemiplimab in the treatment of patients with advanced NSCLC with high PD-L1 expression.

## Data availability statement

The original contributions presented in the study are included in the article/**Supplementary Material**. Further inquiries can be directed to the corresponding author.

## Author contributions

YL participated in study concept and design, constructed the model, did the literature search and the acquisition of data, analyzed the data, and drafted the manuscript. XL conceived and designed the study, constructed the model, did the literature search and the acquisition of data, analyzed the data, and revised the manuscript. TY contributed to interpretation of results and the critical revision of the manuscript for important intellectual content. SG did the critical revision of the manuscript for important intellectual content. XC conceived and designed the study, acquired the funding, did the acquisition of data, revised the manuscript, technical and material support, and approved the final version of the manuscript. All authors read and approved the final manuscript.

## Funding

This work was supported by the National Natural Science Foundation of China (No. 82160763).

## Conflict of interest

The authors declare that the research was conducted in the absence of any commercial or financial relationships that could be construed as a potential conflict of interest.

## Publisher’s note

All claims expressed in this article are solely those of the authors and do not necessarily represent those of their affiliated organizations, or those of the publisher, the editors and the reviewers. Any product that may be evaluated in this article, or claim that may be made by its manufacturer, is not guaranteed or endorsed by the publisher.

## Supplementary material

Supplementary Figure 1 Flowchart of Study Selection.

Supplementary Figure 2 Model Fitting Analysis.

Supplementary Figure 3 Model Schematic for Network Meta-analysis.

Supplementary Figure 4 Risk of Bias Summary.

Supplementary Figure 5 Tornado Diagram of One-Way Sensitivity Analyses of Pembrolizumab Versus Cemiplimab in Order of Magnitude of the Association.

Supplementary Figure 6 Impacts of Key Factors on Incremental Cost-effectiveness Ratio.

Supplementary Table 1 Estimated Parameters and AIC Values from Each Survival Model.

Supplementary Table 2 Associated Costs and Disutility of Grade 3 to 4 Treatment-Related AEs.

Supplementary Table 3 Results of Base-case Analysis Included Platinum-based Chemotherapy.
