# Supplementary material for: Pembrolizumab vs cemiplimab for the treatment of advanced non-small cell lung cancer with PD-L1 expression levels of at least 50%: A network meta-analysis and cost-effectiveness analysis
Source: Front Oncol. 2022 Sep 26;12:878054. doi: 10.3389/fonc.2022.878054 (PMC9549171; doi:10.3389/fonc.2022.878054)
Supplement: Supplementary Figure 1 — Flowchart of Study Selection. [file DataSheet_1.docx]

**Supplementary Content**

**Supplementary Figure 1.** Flowchart of Study Selection

**Supplementary Figure 2.** Model Fitting Analysis

**Supplementary Figure 3.** Model Schematic for Network Meta-analysis

**Supplementary Figure 4.** Risk of Bias Summary

**Supplementary Figure 5.** Tornado Diagram of 1-Way Sensitivity Analyses of Pembrolizumab Versus Cemiplimab in Order of Magnitude of the Association

**Supplementary Figure 6.** Impacts of Key Factors on Incremental Cost-effectiveness Ratio

**Supplementary Table 1.** Estimated Parameters and AIC Values from Each Survival Model

**Supplementary Table 2.** Associated Costs and Disutility of Grade 3 to 4 Treatment-Related Adverse Events

**Supplementary Table 3.** Results of Base-case Analysis Included Platinum-based Chemotherapy

**Supplementary Figure 1.** Flowchart of Study Selection


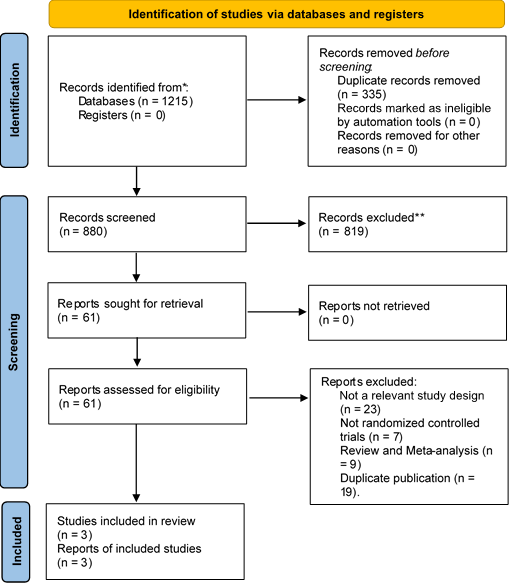


*Consider, if feasible to do so, reporting the number of records identified from each database or register searched (rather than the total number across all databases/registers).

**If automation tools were used, indicate how many records were excluded by a human and how many were excluded by automation tools.

**Supplementary Figure 2.** Model Fitting Analysis

To obtain the best model fit, the following investigations were carried out using pembrolizumab or cemiplimab as the model fit baseline, respectively. Based on AIC and BIC (Supplementary Table 1), log-logistic was used to fit the OS and PFS KM of pembrolizumab and lognormal was chosen to fit the OS and PFS KM of cemiplimab.

(A) Model-fitted versus original KM curves for pembrolizumab.


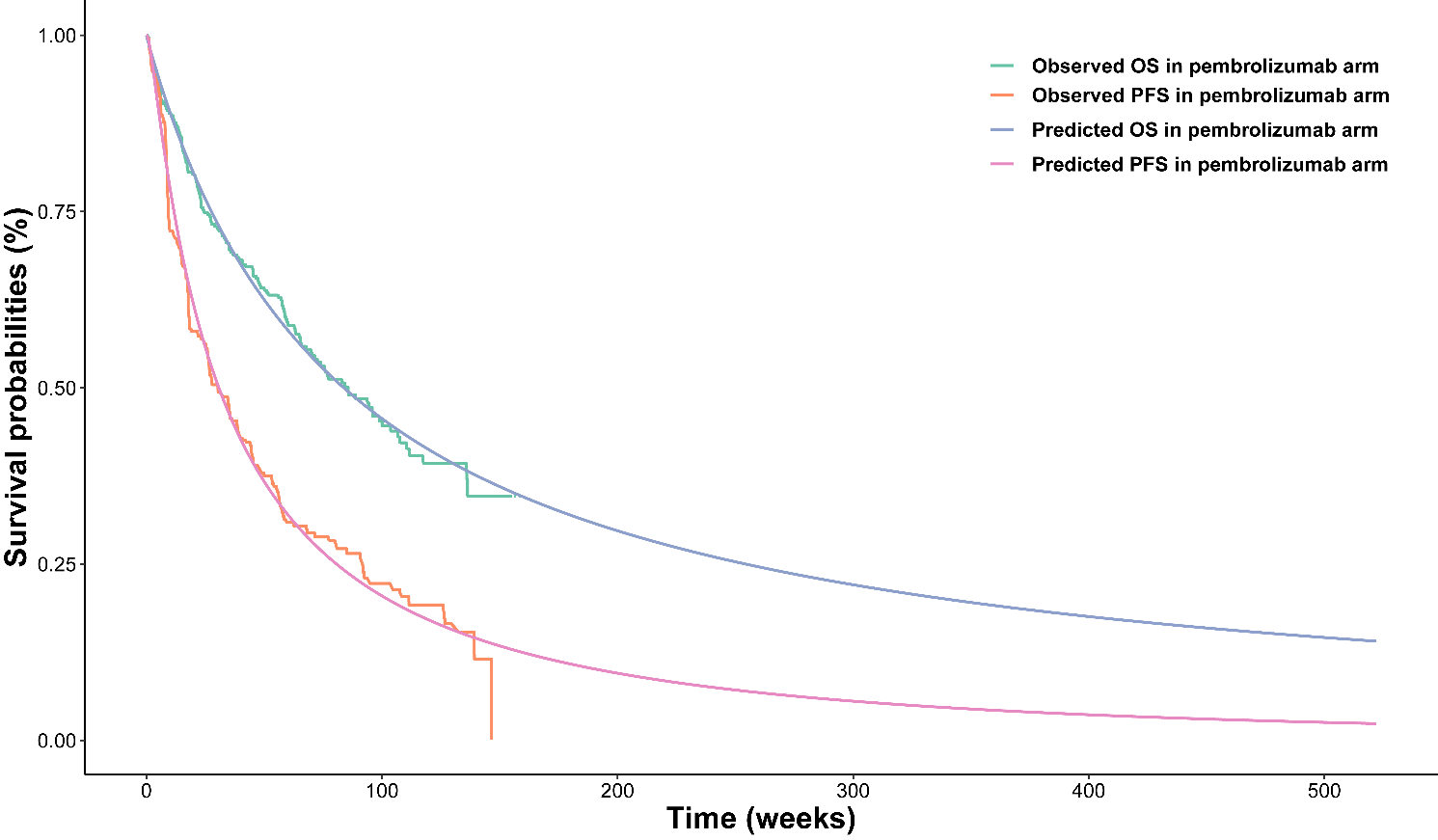


(B) Model-fitted versus original KM curves for cemiplimab.


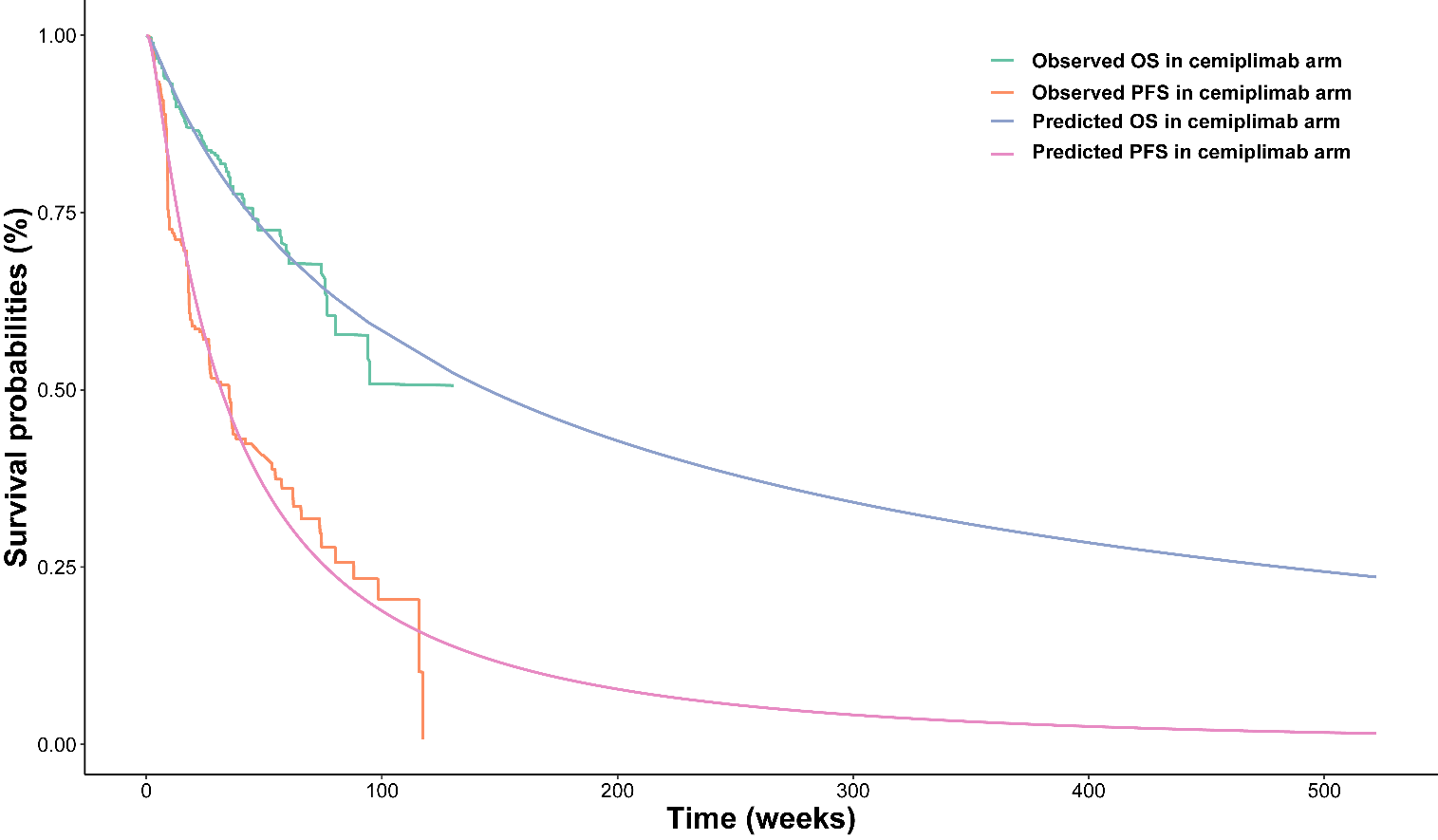


**Supplementary Figure 3.** Model Schematic for Network Meta-analysis


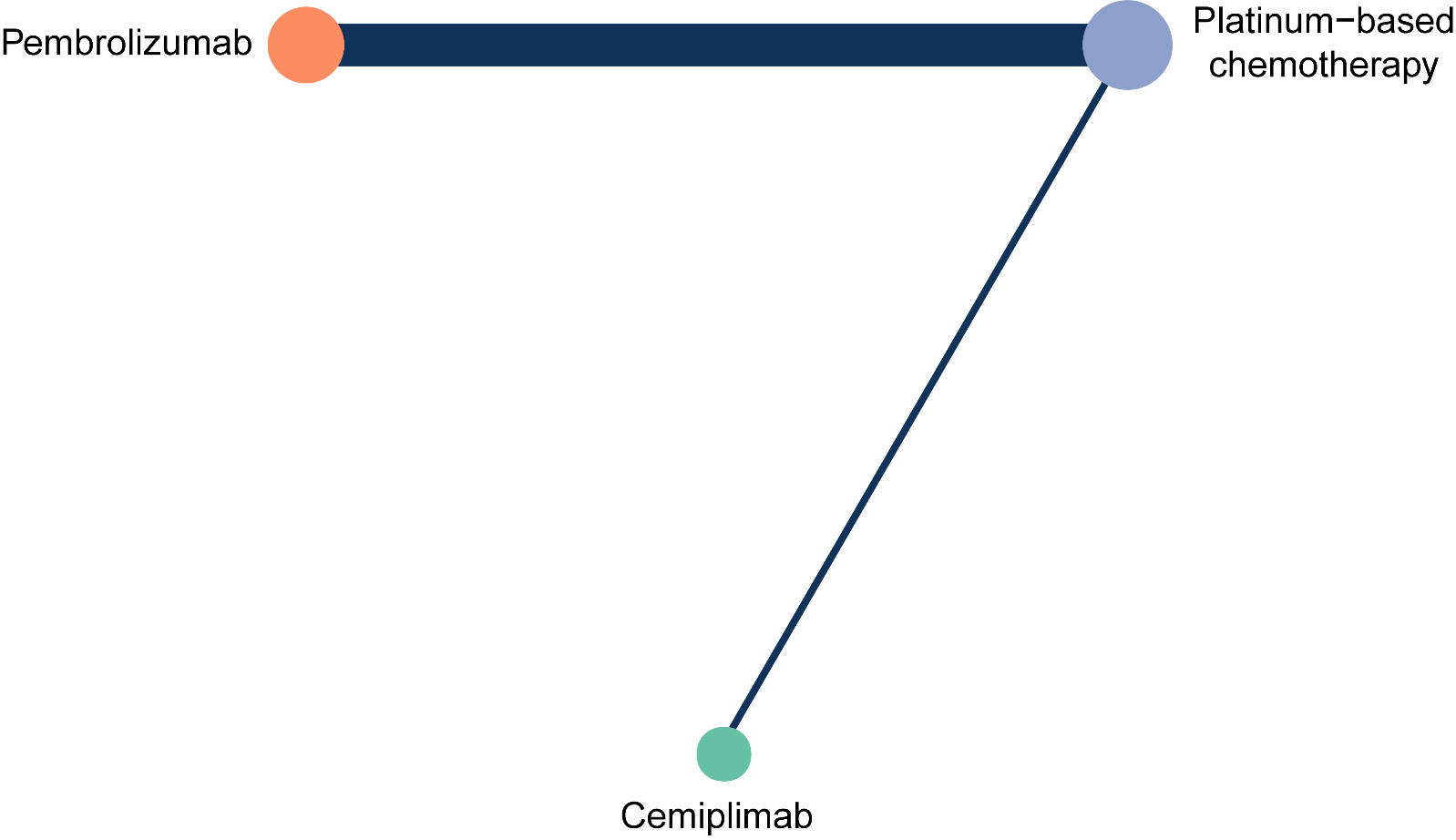


**Supplementary Figure 4.** Risk of Bias Summary


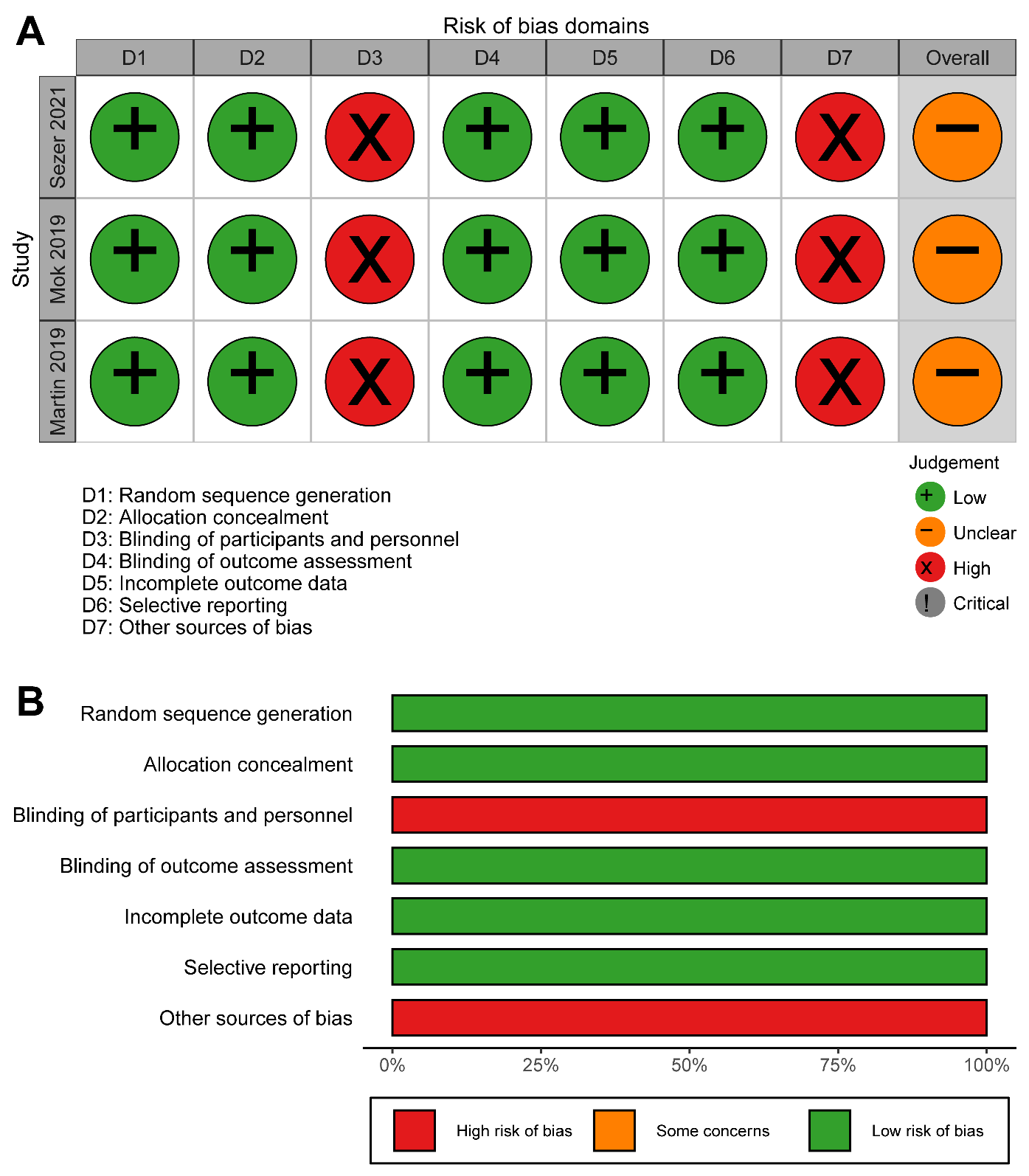


**Supplementary Figure 5.** Tornado Diagram of 1-Way Sensitivity Analyses of Pembrolizumab Versus Cemiplimab in Order of Magnitude of the Association


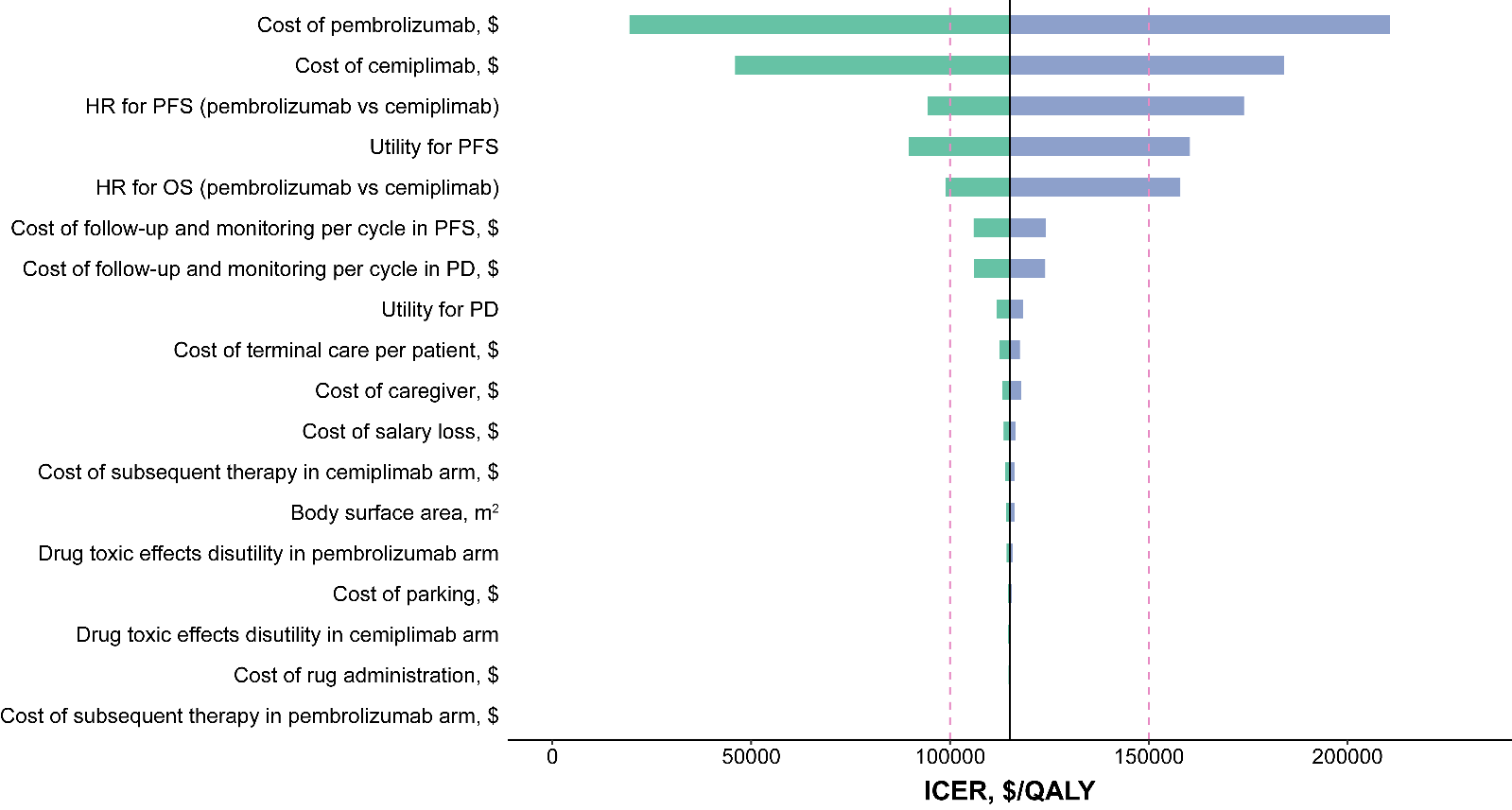


**Supplementary Figure 6.** Impacts of Key Factors on Incremental Cost-effectiveness Ratio

The diagrams show the impacts of key factors on the incremental cost-effectiveness ratio (Pembrolizumab Versus Cemiplimab) for the treatment of advanced NSCLC. (A) represents the impacts of the cost of pembrolizumab; (B) represents the impacts of the cost of cemiplimab. ICER: Incremental cost-effectiveness ratio; QALY: Quality-adjusted life year.

(A) represents the impacts of the cost of pembrolizumab


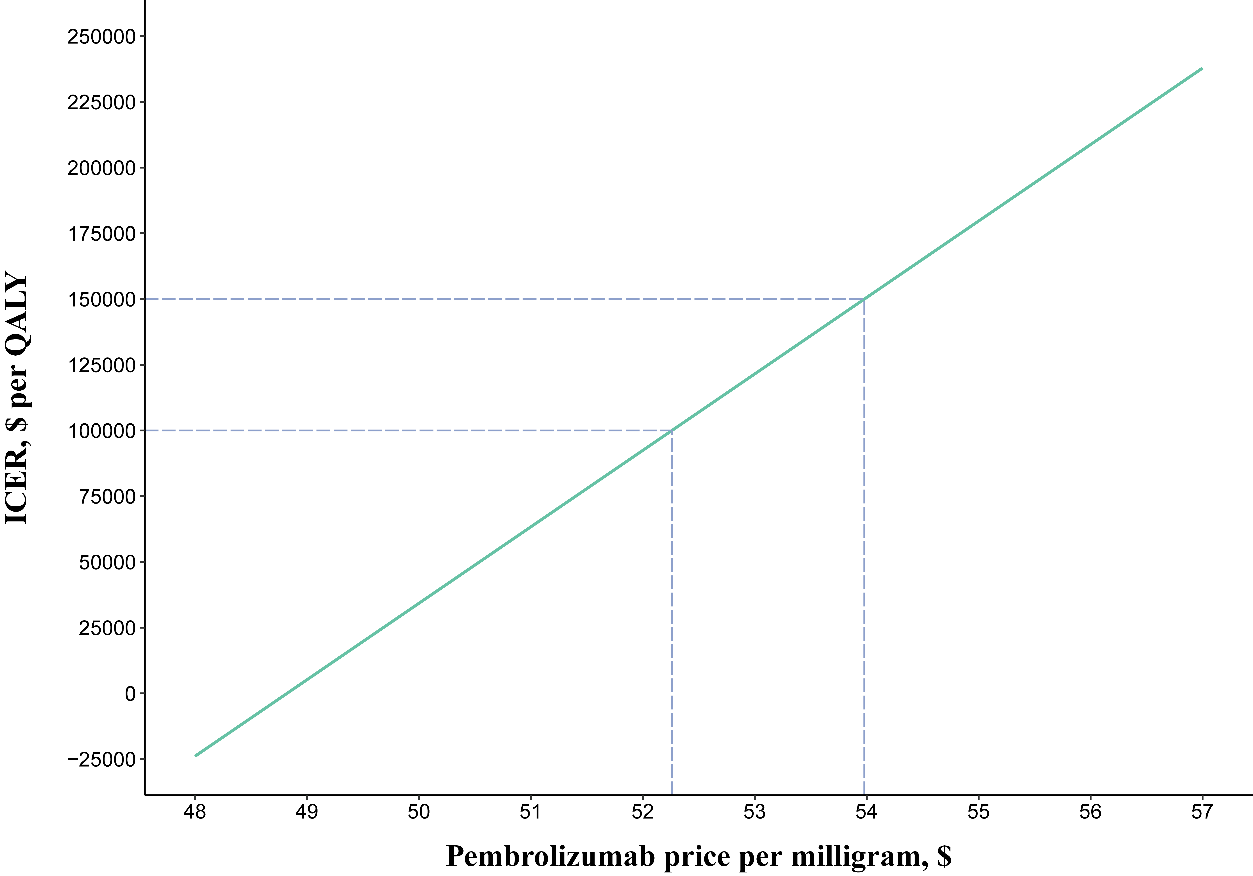


(B) represents the impacts of the cost of cemiplimab


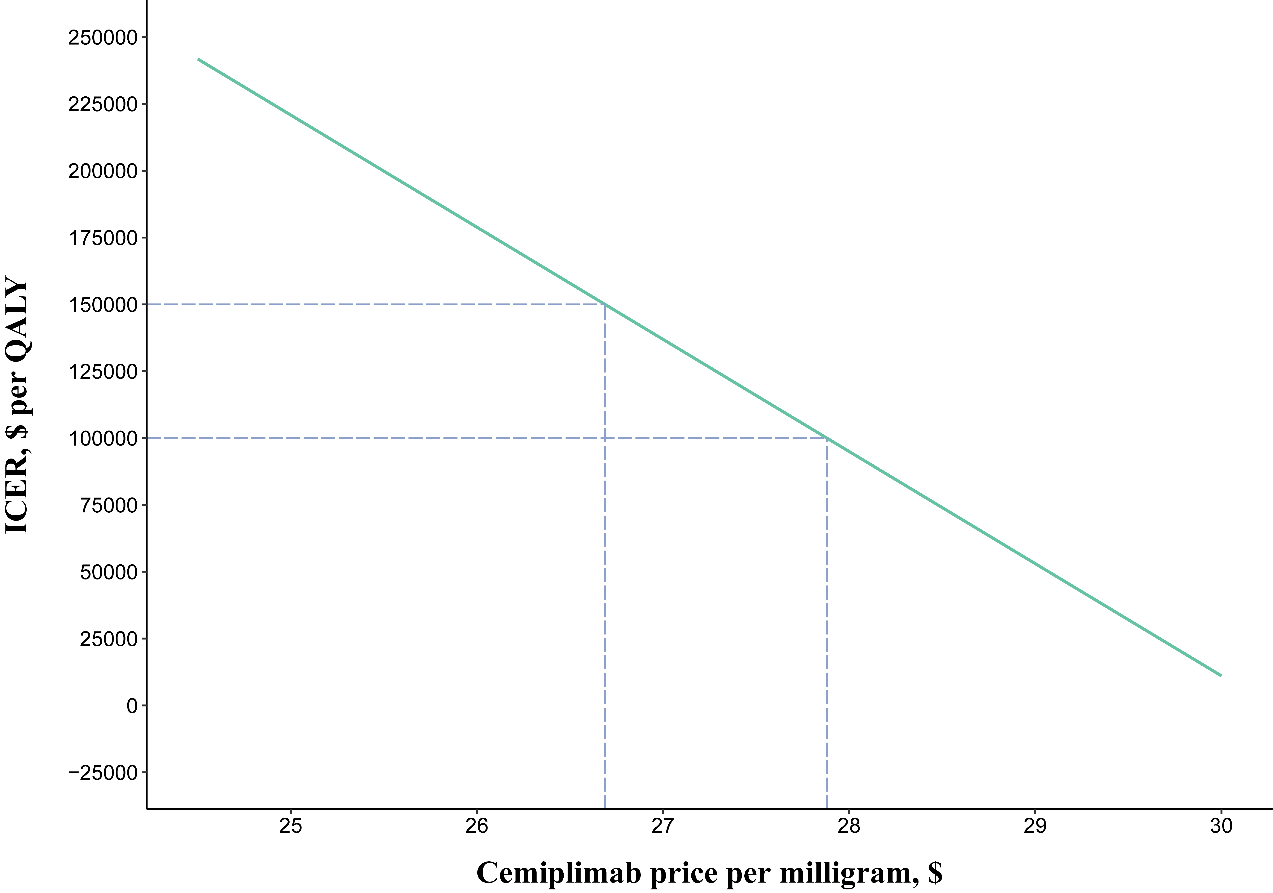


**Supplementary Table 1.** Estimated Parameters and AIC and BIC Values from Each Survival Model

| **Strategies** | **Distributions** | **Parameters** | **est** | **se** | **L95%** | **U95%** | **AIC** | **BIC** |
| --- | --- | --- | --- | --- | --- | --- | --- | --- |
| **Results of OS** | | | | | | | | |
| **Pemblimab** | Exponential | rate | 0.0082657 | 0.0006555 | 0.0070758 | 0.0096557 | 1845.013 | 1848.714 |
|  | WeibullPH | shape | 0.8307687 | 0.0590805 | 0.7226809 | 0.955023 | 1839.688 | 1847.089 |
|  |  | scale | 0.0173308 | 0.0046583 | 0.0102336 | 0.02935 |  |  |
|  | Gamma | shape | 0.798794 | 0.0724884 | 0.6686373 | 0.954287 | 1840.55 | 1847.95 |
|  |  | rate | 0.0058757 | 0.0009964 | 0.0042142 | 0.0081922 |  |  |
|  | Lognormal | meanlog | 4.44166 | 0.124548 | 4.19755 | 4.68577 | 1839.125 | 1846.525 |
|  |  | sdlog | 1.80235 | 0.110651 | 1.59802 | 2.03281 |  |  |
|  | Gompertz | shape | -0.006865 | 0.002603 | -0.011967 | -0.001763 | 1839.596 | 1846.997 |
|  |  | rate | 0.0110201 | 0.0014086 | 0.0085779 | 0.0141576 |  |  |
|  | Log-logistic | shape | 0.98918 | 0.068241 | 0.864079 | 1.13239 | **1838.826** | **1846.227** |
|  |  | scale | 83.81104 | 9.373301 | 67.313802 | 104.35141 |  |  |
|  | Generalized gamma | mu | 4.665823 | 0.192195 | 4.289128 | 5.04252 | 1839.457 | 1850.559 |
|  |  | sigma | 1.548208 | 0.232418 | 1.153577 | 2.07784 |  |  |
|  |  | Q | 0.444966 | 0.341647 | -0.224649 | 1.11458 |  |  |
| **Cemiplimab** | Exponential | rate | 0.0062248 | 0.0007336 | 0.004941 | 0.0078423 | 877.406 | 881.052 |
|  | WeibullPH | shape | 0.9592092 | 0.0974013 | 0.7861031 | 1.1704347 | 879.235 | 886.526 |
|  |  | scale | 0.0073145 | 0.0029411 | 0.003326 | 0.0160858 |  |  |
|  | Gamma | shape | 0.9622857 | 0.1177617 | 0.7570708 | 1.2231269 | 879.307 | 886.598 |
|  |  | rate | 0.0057703 | 0.0015703 | 0.0033849 | 0.0098366 |  |  |
|  | Lognormal | meanlog | 4.97513 | 0.201039 | 4.5811 | 5.36916 | **877.1** | **880.791** |
|  |  | sdlog | 1.78373 | 0.163344 | 1.49067 | 2.13441 |  |  |
|  | Gompertz | shape | -0.003953 | 0.0050687 | -0.013888 | 0.0059813 | 878.777 | 886.068 |
|  |  | rate | 0.0069905 | 0.0012959 | 0.0048608 | 0.0100533 |  |  |
|  | Log-logistic | shape | 1.05998 | 0.106933 | 0.869813 | 1.29172 | 878.541 | 885.832 |
|  |  | scale | 125.68204 | 21.435525 | 89.969999 | 175.56935 |  |  |
|  | Generalized gamma | mu | 5.02187 | 0.234154 | 4.562933 | 5.4808 | 879.397 | 890.333 |
|  |  | sigma | 1.65047 | 0.444338 | 0.973752 | 2.79748 |  |  |
|  |  | Q | 0.18254 | 0.558271 | -0.911652 | 1.27673 |  |  |
| **Results of PFS** | | | | | | | | |
| **Pemblimab** | Exponential | rate | 0.0082657 | 0.0006555 | 0.0070758 | 0.0096557 | 1845.013 | 1848.714 |
|  | WeibullPH | shape | 0.8307687 | 0.0590805 | 0.7226809 | 0.955023 | 1839.688 | 1847.089 |
|  |  | scale | 0.0173308 | 0.0046583 | 0.0102336 | 0.02935 |  |  |
|  | Gamma | shape | 0.798794 | 0.0724884 | 0.6686373 | 0.954287 | 1840.55 | 1847.95 |
|  |  | rate | 0.0058757 | 0.0009964 | 0.0042142 | 0.0081922 |  |  |
|  | Lognormal | meanlog | 4.44166 | 0.124548 | 4.19755 | 4.68577 | 1839.125 | 1846.525 |
|  |  | sdlog | 1.80235 | 0.110651 | 1.59802 | 2.03281 |  |  |
|  | Gompertz | shape | -0.006865 | 0.002603 | -0.011967 | -0.001763 | 1839.596 | 1846.997 |
|  |  | rate | 0.0110201 | 0.0014086 | 0.0085779 | 0.0141576 |  |  |
|  | Log-logistic | shape | 0.98918 | 0.068241 | 0.864079 | 1.13239 | **1838.826** | **1846.227** |
|  |  | scale | 83.81104 | 9.373301 | 67.313802 | 104.35141 |  |  |
|  | Generalized gamma | mu | 4.665823 | 0.192195 | 4.289128 | 5.04252 | 1839.457 | 1850.559 |
|  |  | sigma | 1.548208 | 0.232418 | 1.153577 | 2.07784 |  |  |
|  |  | Q | 0.444966 | 0.341647 | -0.224649 | 1.11458 |  |  |
| **Cemiplimab** | Exponential | rate | 0.0196969 | 0.0015872 | 0.0168193 | 0.023067 | 1519.606 | 1523.252 |
|  | WeibullPH | shape | 0.9947856 | 0.0642291 | 0.8765385 | 1.1289845 | 1521.6 | 1528.89 |
|  |  | scale | 0.0200783 | 0.0050095 | 0.0123127 | 0.0327417 |  |  |
|  | Gamma | shape | 1.0495449 | 0.0967251 | 0.876103 | 1.2573233 | 1521.333 | 1528.624 |
|  |  | rate | 0.0210503 | 0.003121 | 0.015742 | 0.0281488 |  |  |
|  | Lognormal | meanlog | 3.45945 | 0.0917086 | 3.2797 | 3.63919 | **1501.711** | **1509.002** |
|  |  | sdlog | 1.29574 | 0.0775023 | 1.15241 | 1.45691 |  |  |
|  | Gompertz | shape | -0.005874 | 0.0039328 | -0.013583 | 0.001834 | 1519.238 | 1526.529 |
|  |  | rate | 0.0226064 | 0.0026826 | 0.0179153 | 0.028526 |  |  |
|  | Log-logistic | shape | 1.29715 | 0.0851189 | 1.14061 | 1.47519 | 1510.256 | 1517.547 |
|  |  | scale | 31.42635 | 2.8581467 | 26.29537 | 37.55852 |  |  |
|  | Generalized gamma | mu | 3.189461 | 0.1962696 | 2.80478 | 3.574142 | 1501.09 | 1512.027 |
|  |  | sigma | 1.369154 | 0.0853824 | 1.21163 | 1.547157 |  |  |
|  |  | Q | -0.512467 | 0.3151305 | -1.13011 | 0.105177 |  |  |

**Abbreviation:** AIC, Akaike information criterion; BIC, Bayesian information criterion

**Supplementary Table 2.** Associated Costs and Disutility of Grade 3 to 4 Treatment-Related Adverse Events

| **Adverse Event^a^** | **No. of patients (%)^b^** | **Costs in 2021 USD** | **Reference** | **Disutility** | **Reference** |
| --- | --- | --- | --- | --- | --- |
| **Pembrolizumab** |  |  |  |  |  |
| Fatigue, asthenia | 6 (0.94%) | 10549.09 | Konidaris et al, 2020 | 0.2880 | Nafees et al, 2017 |
| Rash, Pruritus | 5 (0.79%) | 6006.22 | Konidaris et al, 2020 | 0.1560 | Nafees et al, 2017 |
| Decreased appetite, nausea, vomiting | 5 (0.79%) | 18301.44 | Wong et al, 2018 | 0.2040 | Nafees et al, 2017 |
| Anaemia | 4 (0.63%) | 8007.96 | Konidaris et al, 2020 | 0.0720 | Freeman et al, 2015 |
| Diarrhoea | 5 (0.79%) | 17880.16 | Wong et al, 2018 | 0.2160 | Nafees et al, 2017 |
| Neutropenia | 1 (0.16%) | 18606.85 | Wong et al, 2018 | 0.3480 | Nafees et al, 2017 |
| Infection | 20 (3.14%) | 17202.21 | Wong et al, 2018 | 0.3480 | Nafees et al, 2017 |
| Weighted averaged^c^ |  | 1051.76 |  | 0.0192 |  |
| **Cemiplimab** |  |  |  |  |  |
| Fatigue, asthenia | 3 (0.85%) | 10549.09 | Konidaris et al, 2020 | 0.2880 | Nafees et al, 2017 |
| Rash, Pruritus | 3 (0.85%) | 6006.22 | Konidaris et al, 2020 | 0.1560 | Nafees et al, 2017 |
| Decreased appetite, nausea, vomiting | 1 (0.28%) | 18301.44 | Wong et al, 2018 | 0.2040 | Nafees et al, 2017 |
| Anaemia | 2 (0.56%) | 8007.96 | Konidaris et al, 2020 | 0.0720 | Freeman et al, 2015 |
| Diarrhoea | 1 (0.28%) | 17880.16 | Wong et al, 2018 | 0.2160 | Nafees et al, 2017 |
| Neutropenia | 2 (0.56%) | 18606.85 | Wong et al, 2018 | 0.3480 | Nafees et al, 2017 |
| Infection | 1 (0.28%) | 17202.21 | Wong et al, 2018 | 0.3480 | Nafees et al, 2017 |
| Weighted averaged^c^ |  | 440.22 |  | 0.0083 |  |
| **Platinum-based chemotherapy** | |  |  |  |  |
| Fatigue, asthenia | 4 (1.17%) | 10549.09 | Konidaris et al, 2020 | 0.2880 | Nafees et al, 2017 |
| Rash, Pruritus | 0 (0%) | 6006.22 | Konidaris et al, 2020 | 0.1560 | Nafees et al, 2017 |
| Decreased appetite, nausea, vomiting | 7 (2.05%) | 17405.97 | Wong et al, 2018 | 0.2040 | Nafees et al, 2017 |
| Anaemia | 51 (14.91%) | 8150.39 | Konidaris et al, 2020 | 0.0720 | Freeman et al, 2015 |
| Diarrhoea | 6 (1.75%) | 17005.30 | Wong et al, 2018 | 0.2160 | Nafees et al, 2017 |
| Neutropenia | 35 (10.23%) | 18774.14 | Wong et al, 2018 | 0.3480 | Nafees et al, 2017 |
| Infection | 0 (0%) | 23962.41 | Wong et al, 2018 | 0.3480 | Nafees et al, 2017 |
| Weighted averaged^c^ |  | 3910.04 |  | 0.0577 |  |

^a^Our analysis only included and evaluated grade 3-4 treatment-related adverse events.

^b^Number within treatment arm: pembrolizumab (N=790), cemiplimab (N=355).

^c^Calculated as an average cost of toxicity using the weighted frequency of occurrence. This value was used in the base-case model.

**Supplementary Table 3.** Results of Base-case Analysis Included Platinum-based Chemotherapy

| **Drug** | **Cost** | **QALYs** | **Life-years** | **ICER (Platinum-based chemotherapy as a reference standard)** | **ICER (Cemiplimab as a reference standard)** |
| --- | --- | --- | --- | --- | --- |
| Pembrolizumab | 282613 | 0.920 | 2.394 | 175442 | 114246 |
| Cemiplimab | 271957 | 0.826 | 2.637 | 211130 | / |
| Platinum-based chemotherapy | 238187 | 0.666 | 2.177 | / | / |
